# Why is Antonovsky's sense of coherence not correlated to physical health? Analysing Antonovsky's 29-item sense of coherence scale (SOC-29)

**DOI:** 10.1100/tsw.2005.89

**Published:** 2005-09-14

**Authors:** Trine Flensborg-Madsen, Søren Ventegodt, Joav Merrick

**Affiliations:** ^1^ The Quality of Life Research Center, Teglgårdstråde 4-8, DK-1452 Copenhagen K, Denmark; ^2^ Nordic School of Holistic Medicine, Teglgårdstråde 4-8, DK-1452 Copenhagen K, Denmark; ^3^ Quality of life Research Clinic, Teglgårdstråde 4-8, DK-1452 Copenhagen K, Denmark; ^4^ National Institute of Child Health and Human Development, Faculty of Health Sciences, Ben Gurion University of the Negev, Beer-Sheva, Israel; ^5^ Center for Multidisciplinary Research in Aging, Faculty of Health Sciences, Ben Gurion University of the Negev, Beer-Sheva, Israel; ^6^ Division of Pediatrics, Faculty of Health Sciences, Ben Gurion University of the Negev, Beer-Sheva, Israel; ^7^ Office of the Medical Director, Division for Mental Retardation, Ministry of Social Affairs, Jerusalem, Israel

**Keywords:** Antonovsky, sense of coherence, public health, human development, Denmark

## Abstract

We have previously concluded that the use of the Antonovsky sense of coherence (SOC) scale was unable to document a predicted strong association between SOC and physical health. By way of statistical methods, numerous studies have investigated the validity, reliability and applicability of the SOC scale with positive results. However, this paper analyses whether the questions in the SOC scale actually represent the universe of factors necessary to describe the phenomenon of SOC, which we believe is an important supplement to the statistically means of investigating validity and reliability. In this paper we explore the *idea*, *the concepts*, *the theory* and *the operationalisation* behind the SOC Scale. The conclusions are: 1) it seems that Antonovsky's basic idea of coherence, for which he coined the term sense of coherence, as the basis for the highly popular salutogenic orientation is outstandingly good, in spite of the lack of statistical evidence; 2) the chosen key explanatory concepts of *comprehensibility*, *manageability*, and *meaning*, seems to be a fair, although mental, conceptualisation of this idea; 3) Antonovsky's theory was unfortunately much less clear, as Antonovsky assumed predictability to be very important for the sense of coherence, especially for comprehensibility and manageability. This notion of predictability leaves its footprints in his operationalization of SOC into the SOC Scale. Our analysis convinced us that the SOC scale is unlikely to be a fair materialization of the idea of coherence and thus unlikely to measure SOC correctly.

## INTRODUCTION

The Sense of Coherence Scale invented by Aaron Antonovsky (1923-1994), from the Faculty of Health Sciences at the Ben Gurion University in the Negev, intended to measure an aspect that was of crucial and causal importance to health: the *sense of coherence* (SOC). Antonovsky's idea of coherence between life inside and the world outside as the true source of health has gained popularity worldwide, and the SOC Scale has been used in several studies to verify the hypothesis that the degree of a person's SOC is strongly associated with his health. Several studies in many cultures have shown associations between SOC and a variety of problems in the area of health and well-being. However, most of these studies showed only modest relations to physical symptoms[1-8], as described in a previous paper. We concluded that the SOC scale can only serve as a predictor for health that is measured by incorporating psychological aspects, while it is not capable of explaining health measured only by means of physical terms[9].

In this paper, we explore the idea, the concepts, the theory and the operationalization behind the SOC scale. We believe that two fundamental problems exist in relation to the SOC scale. The first problem is that the questions in the SOC scale are both of a mental and of an emotional nature. We believe that it is the emotionality and not the mentality that determines the physical health, which is the reason why it has not been possible to prove a strong association between SOC and physical health. We believe this is due to the fact that the scales mixes up the two concepts, a topic which we have discussed in depth elsewhere[10-12]. The other problem is that the scale contains a notion of predictability, with the intention that a person scores a high sense of coherence if his life seems predictable, which we believe is an incorrect assumption.

## THE IDEA BEHIND THE SENSE OF COHERENCE SCALE

The shaping of Antonovsky's ideas took place in the 1970s, while he was analysing data from a study about adaptation to menopause of women from different ethnic groups and comparing emotional health ratings of a group of concentration camp survivors to those of a control group. He found that 51 % of the control group, as compared to 29 % of the survivors, were in good overall emotional health. The same consistency was found concerning physical health[13]. Instead of looking at the fact that 51 % was much more than 29 %, he focused on the fact that 29 % of the women who had been in a concentration camp were categorized as having a reasonable mental health. Antonovsky expressed his thoughts: " *To have gone through the most unimaginable horror of the camp, followed by years of being a displaced person, and then to have re-established one's life in a country which witnessed three wars…and still be in reasonable health. This, for me, was the dramatic experience which consciously set me on the road…*"[13].

In the western world, illness is not an infrequent event, since a majority of the population has some pathological condition. In Denmark, for example, we found that 94% of the population suffered from one or more health problems[14,15]. The most common approach to disease is the pathological orientation which tries to explain why people get sick. In contrast to this predominant paradigm of pathogenesis in the western world, Antonovsky introduced in 1979 the concept of salutogenesis (Latin: salus meaning health, or well-being)[16]. The consequences of adopting a salutogenic paradigm can be exemplified largely by six facets[17]:

- None is categorized as healthy or diseased; rather we are all somewhere between the two poles of total wellness and total illness.
- The search for the mystery of health is not limited to particular disease entities, but rather the generalized factors that are involved.
- Attention is turned away from the potential pathogens and focused on the resources that are valuable in coping with the pathogens and stressors.

- The question is not focused on how to eradicate particular stressors, but on how to learn to live well with the stressors that exist.
- The paradigm opens the way for cooperation between different groups of scientists and practitioners.
- The focus is on deviants rather than on high-risk groups. The focus is on “the symptoms of wellness”.

The salutogenic orientation seeks to understand why people are located toward the positive end of the disease continuum, or why they are moving towards this end. This is contrary to the pathological orientation, which seeks to explain the moving towards disease and investigates life stressors ranging from microbiological to societal-cultural levels. As Antonovsky wrote, stressors are omnipresent in the nature of the human existence, but nevertheless many people, even with a high stressor load, do not get sick. “*Barring stressors that directly destroy the organism, people’s health outcomes are unpredictable. This is the mystery the salutogenic orientation seeks to unravel*”[10]. Antonovsky proposed that coming across the various stressors in life resulted in tensions that one must deal with. The capability of managing the tension determines whether the outcome will be pathological, neutral or salutary. Antonovsky’s health science, therefore, centers on the factors that determine the individual’s capability in this respect. Therefore, the studying of factors that determine the tension management was the key idea in the health science of Antonovsky. The sense of coherence, according to Antonovsky, is of crucial importance to our health: *“My hypothesis, then, is that the strength of the SOC has direct physiological consequences and, through such pathways, affects health status”*[13].Antonovsky operationalized the SOC in terms of a 29-item and also a 13-item self-report measure on a seven point semantic differential scale. These scales have been used in at least 33 languages in 32 countries (including some with different versions of the scale)[18]. However, an intensive search for studies with the aim to verify the hypothesis of an association between SOC and physical health showed that it is complicated to prove. In a previous paper, we reviewed the available scientific publications concerning the association between the sense of coherence, measured with the scales SOC-29 or SOC-13, and different aspects of health[1]. The majority of the published studies measured the association with psychological health, or incorporated psychological health, in their health measures. We found that SOC was highly associated with psychological measures[19-29], with health measures that incorporate psychological measures[30-33], stress[34-40] and behaviours[41-46]. It was concluded that the SOC scale was unable to show a strong association between SOC and physical health, as Antonovsky had claimed, because only a few modest correlations were found with physical health (correlations ranging from 0.17 - 0.51)[2-9]. About half of the studies showed insignificant correlations, and in addition to this we must suspect a certain amount of publication bias[1]. The results from the above study suggest that only 4%-20% of the physical health can be explained from SOC, with SOC measured by the present scale. This is not astonishing, since in a previous study we found that global quality of life explained 25% of the physical self-evaluated health[14,15].

Since the overall evaluation of the published studies of associations with SOC seems quite complex and divergent, we began to suspect that the SOC scale simply does not measure SOC well enough to demonstrate the strong association to health as predicted by Antonovsky. We believe that the idea of sense of coherence is in itself outstanding and therefore undertook an analysis of Antonovsky’s work from conceptualization, to theory and operationalization of the questionnaire, and our analysis seems to reveal the reason for the disappointing empirical findings and also brings forward several suggestions for improvement of the SOC scale.

## THE CONCEPTUALIZATION OF THE IDEA

It seems that Antonovsky was not interested in the study of particular diseases, but rather had a focus on factors that could describe the ability to manage tension. At first, these factors were described by the concept of *generalized resistance resources* (GRRs), defined as money, ego strength, cultural stability, social support and the like (any phenomenon that is effective in combating a wide variety of stressors). After creating the concepts of GRR, Antonovsky felt that something was lacking in order to describe his idea in a profound way. He wanted to find out *why* a certain phenomenon could be identified as a GRR – what was the reason that a phenomenon served as a GRR. This led to the development of the sense of coherence concept. Antonovsky proposed that what was common to all the factors included in the GRR was that they facilitated making sense out of the countless stressors with which we are constantly bombarded. He formulated the SOC as:

"…a global orientation that expresses the extent to which one has a pervasive, enduring though dynamic, feeling of confidence that one's internal and external environments are predictable and that there is a high probability that things will work out as well as can reasonably be expected"[13].

In defining the concept, Antonovsky made the presumption that occurrences have to be predictable in order for a person to have a high sense of coherence. This is a significant statement in his theory, since it can reasonably be debated whether it is correct to include predictability in sense of coherence. We support the idea of Occam's Razor (Ockham's Razor, or any of several other spellings, is a principle attributed to the 14th century English logician and Franciscan friar, William of Ockham (1285-1349), which forms the basis of methodological reductionism, also called the principle of parsimony, or law of economy.) In its simplest form, Occam's Razor states that one should make no more assumptions than needed. When multiple explanations are available for a phenomenon, the simplest version is preferred and only the absolutely necessary assumptions should be included in explaining a phenomenon. Therefore, we do not think that predictability is a concept that is rational to include in SOC. Lack of predictability is not necessarily an unpleasant, or unhealthy, phenomenon in life, since we believe that disruption in life is a constant in human experience and unpredictability is a generic feature that is definitive of the human condition. We believe that uncertainty is what makes life and death matter in the first place. Lack of predictability can be an expression of a very meaningful life and provide a state of initiative, energy and positive attitudes. We believe that the philosophy should not presuppose that unpredictability is something that is merely negative.

## FROM THEORY TO CONCEPTS

The core concept of the salutogenic model of Antonovsky, the SOC, was described as: *"The sense of coherence is a global orientation that expresses the extent to which one has a pervasive, enduring though dynamic, feeling of confidence that (1) the stimuli deriving from one's internal and external environments in the course of living are structured, predictable and explicable [comprehensibility]; (2) the resources are available to one to meet the demands posed by these stimuli [manageability]; and (3) these demands are challenges, worthy of investment and engagement [meaningfulness]"*[13].

Hence, the SOC was described in terms of three dimensions: Comprehensibility, manageability and meaningfulness. *Comprehensibility* is described as the extent to which one perceives confronted stimuli as making cognitive sense as information in order, consistency, structure and clarity, rather than as chaotic, disordered, random, accidental, or inexplicable "noise". A high sense of comprehensibility is when a person expects that the stimuli met in the future will be predictable, or that when they do come as surprises, they will be ordered and explicable. *Manageability* is described as the extent to which one perceives the resources adequate to meet the demands posed by the stimuli that bombard him. A high degree of manageability would mean that one will not feel victimized or feel that life is unfair, but rather feel able to cope without grieving endlessly. *Meaningfulness* is described as the extent to which life makes sense and that at least some of the problems and demands posed by living are worth the investment of energy. A high degree of meaningfulness will make challenges welcome rather than a burden that one would very much prefer to be without. When unhappy experiences occur, one will be determined to seek meaning in them and do the best to overcome them.

We classify the theory, including the three aspects behind the "sense of coherence", as being undeniably well argued and correct. However, predictability is present in the theory as was also the case in the conceptualisation. The aspect of predictability is presented within the dimension of comprehensibility where it is argued that being predictable is essential for comprehension of life. However, we do not consider predictability to have any relation to comprehensibility. The reason for our divergence with Antonovsky seems to originate in the understanding of the influence of time. Antonovsky claimed that the present state of comprehensibility is dependent on expectations for the future, which indicates a specific time perspective. He also claimed that these expectations ahead of time had to be set with limited boundaries, since they have to be predictable: for things to be predictable they have to resemble something that is already there. (This will not always be the case, since it is possible to expect things that are different from what they are now, but in everyday terms predictability usually implies that life is pretty much floating along the same road that it always has.) The authors claim that the fact of life going along the same road is not necessarily something that heightens our comprehension. Instead, unpredictability can be seen as an advantage compared to predictability, since it illustrates a will to change course and to live life as fully as possible. A life that is unpredictable is a life that encounters changes and new aspects, which might even improve comprehension. Therefore, the concept of predictability should not be built into the definition of comprehension and, especially, its absence should not be defined as something negative. It can be claimed that people with very unpredictable lives have chosen the unpredictability, because they are capable of dealing with the stimuli through life and therefore in fact are very strong people who should be defined as having a high sense of comprehensibility and as a consequence a high sense of coherence. Apart from the aspect of predictability, the rest of the theory seems appealing as a vehicle to describe the idea of the salutogenic orientation.

Antonovsky stated that it *is* indeed possible to have a strong SOC and at the same time not see the entire world as coherent. This is due to the fact that all people set boundaries, and what is beyond these boundaries simply does not matter to us, whether comprehensible, manageable or meaningful. This boundary notion suggests that it is not necessary to feel that all of life is highly comprehensible, manageable and meaningful in order to have a strong SOC. Rather, it is crucial whether there are spheres of life that are of subjective importance to life and whether these spheres are seen as being comprehensible, manageable and meaningful. Having stated this, Antonovsky made the significant point of defining four spheres from which it is not possible to narrow the boundaries – one's inner feelings, one's immediate interpersonal relations, one's major activities and existential issues – and yet maintain a strong SOC. The reason for this notion, according to Antonovsky, is that too much of our energies and our selves are inevitably bound up on these spheres.

## OPERATIONALIZATION, THE CONCEPT

In order to operationalize the concept of sense of coherence, Antonovsky constructed his SOC scale. The scale consists of 29 items (SOC-29), but a shorter version (SOC-13) has also been constructed. The 29-item questionnaire consists of 11 questions on comprehensibility, 10 on manageability and eight on meaningfulness. The questions are all interrelated to one of the four spheres and the answers should be given on a seven-point semantic differential scale with two anchoring phrases.

The authors have critically reviewed the questions found in the SOC scale. We found that some of the questions are not in accordance with the idea of sense of coherence. Out of the 11 questions in relation to comprehensibility, the following five were found not to be appropriate to include:

**(1) "When you talk to people, do you have the feeling that they don't understand you?"**
*(Reply: Never have this feeling – Always have this feeling)*
**(10) "In the past ten years your life has been?"**
*(Reply: Full of changes without your knowing what will happen next – Completely consistent and clear)*

**(17) "Your life in the future will probably be":**
*(Reply: Full of changes without your knowing what will happen next – completely consistent and clear)*
**(19) "Do you have very mixed-up feelings and ideas?"**
*(Reply: Very often – very seldom or never)*
**(21) Does it happen that you have feelings inside you would rather not feel?"**
*(Reply: Very often – very seldom or never)*

Question (1) seems to be formulated incorrectly. Since the question seeks to find out about the replier's sense of comprehensibility, a more correct question would be whether you feel that *you understand other people.* We believe that it is possible to have a high sense of coherence even though you stand out from of the rest of the group, as long as you understand them. Questions (10), (17), (19) and (21) are all operationalized in the shape of predictability, implying that lack of predictability is something negative for comprehension. The questions illustrate that it is better to have a life that is consistent and clear rather than a life that is full of changes. It can be argued that the fact of having mixed-up feelings and ideas and sometimes feeling that you would rather not have them indicates that one is not afraid to follow true beliefs, even though the possibility is there for things to go wrong. The above-written questions reflect whether a person has a dynamic life and does not reveal aspects of comprehensibility.

Of the ten questions in relation to manageability, the following three were found not to be appropriate:

**(6) "Has it happened that people whom you counted on disappointed you?**
*(Reply: Never happened – always happened)*
**(20) "When you do something that gives you a good feeling:**
*(Reply: It's certain that you'll go on feeling good – it's certain that something will happen to spoil the feeling)*
**(25) "Many people – even those with a strong character – sometimes feel like sad sacks (losers) in certain situations. How often have you felt this way in the past?"**
*(Reply: Never – very often)*

The above questions were found not to describe manageability, but instead, the aspect of predictability. They all describe the aspects of a dynamic life with up and downs in relation to a life in which everything is straight and nice. The above-mentioned questions about the ability to manage things are concerned with the thought of staying along the same line of happiness and never running into awful experiences.

All eight questions concerning meaningfulness were considered to be very appropriate. In conclusion, eight of the 29 questions were considered to be improper to include in a measure of the sense of coherence and four of these questions are also included in the 13-item scale.

## DISCUSSION

This paper is entirely focused on a conceptual analysis and a critique of wording – something we believe to be an important supplement to the statistical means of investigating validity and reliability. The SOC scale has been evaluated in various studies according to validity[15,19,26], reliability[47] and applicability[48] with positive results. However, one study concluded that the scale was measuring something other than sense of coherence, namely emotionality, and that it lacked construct validity[16]. The SOC measure has been used in at least 33 languages in 32 countries and is therefore a widely used measure[15]. Besides its association to health measures, the concept of SOC has been evaluated in terms of numerous other factors like structural sources[49], its relation to meaning, locus of control, learned helplessness and hardiness[50], aging[51] and its relation to modern physics[52]. The concept of SOC has, among other things, been used in the discussion of the promotion of different issues like how to use the potential of physician-patient-communication to promote health[53] and the construction of the patient personality profile[54].

Despite the extensive use of the SOC scale and despite the fact that diverse studies have proven its reliability and validity to be very good, we still consider the SOC scale to be deficient. The reliability can be measured in terms of two facets: the test-retest reliability and the internal reliability. However, neither of these inform about the meaning of the scale. The first is an expression of the scale's ability to produce the same result every time, while the second is an expression of whether the items in the scale are measuring the same thing, which in this case is SOC. The internal reliability is most often measured in terms of Cronbach's alpha, where a high value means that all the items are contributing to the concept that is measured. However, a high alpha value is not an expression of a theoretically meaningful scale. The value of alpha increases with the number of items and also increases if the items incorporated resemble each other. Therefore it is not a piece of art to create a high value of Cronbach's alpha, since it does not inform us whether the scale is measuring what it is supposed to measure. The validity of the scale has been examined in various studies in terms of face validity, consensual validity, construct validity, criterion validity and predictive validity with different results[15]. One of the leading researchers in the field, Bengt Lindström, from the Nordic School of Public Health in Sweden, has recently declared that the SOC instrument is reliable, valid, feasible and cross culturally applicable[15]. Nevertheless, he also mentions that the structure of the SOC concept is still not completely clear[15].

In this paper we looked at the validity of the questions in the scale. We investigated whether the questions represented the universe of factors necessary to describe the phenomenon of SOC and if they are actually measuring the original idea of sense of coherence. Our conclusion was that eight out of 29 questions were improper to include in a SOC scale. With empirical data at hand it may be possible by a factor-analytic approach to verify that the items we see as misplaced indeed do not belong to the SOC sphere, but vary fairly independently from the genuinely SOC-related items.

## CONCLUSION

Aaron Antonovsky coined the coherence between life inside and the world outside as the "sense of coherence" and he hypothesized this coherence to be the major predictor of health. Unfortunately, his sense of coherence scale has not been able to document the predicted strong correlations between SOC and physical health. This paper suggests that while the idea of sense of coherence is sound, the conceptualization, the theory and the operationalization of the idea by Antonovsky were inappropriate insofar as a strange concept of predictability (indicating a preference of Antonovsky for a "non-dynamic", stable and predictable life) has been built into the scale as a fixed prerequisite for attaining a strong sense of coherence. We suggest that a better SOC scale can be constructed without the concept of predictability and that eight of the questions in the 29-item SOC scale are improper to include.

## ACKNOWLEDGEMENTS

This study was supported by grants from IMK Almene Fond. The quality of life research was approved by the Copenhagen Scientific Ethical Committee under number (KF)V.100.2123/91.

## BIOSKETCHES

**Trine Flensborg-Madsen**, **BScA**, is a research assistant at the Quality of Life Research Center in Copenhagen, Denmark. She is at this time finishing her master degree in Public Health at the Department of Public Health, University of Copenhagen. E-mail: tfm@livskvalitet.org

**Søren Ventegodt, MD,** is the director of the Quality of Life Research Center in Copenhagen, Denmark. He is also responsible for a Research Clinic for Holistic Medicine in Copenhagen and noted as a popular speaker throughout Scandinavia. He is a teacher at Nordic School of Holistic Medicine. He has published numerous scientific or popular articles and a number of books on holistic medicine, quality of life and quality of working life. His most important scientific contributions are the comprehensive SEQOL questionnaire, the very short QoL5 questionnaire, the integrated QOL theory, the holistic process theory of healing, the life mission theory, the theory of human character, the theory of existential coherence, and the Danish Quality of Life Research Survey, 1991-94 in cooperation with the University Hospital of Copenhagen and the late professor of pediatrics, Bengt Zachau-Christiansen, MD, PhD. E-Mail: ventegodt@livskvalitet.org. Website: www.livskvalitet.org

**Joav Merrick, MD, DMSc,** is professor of child health and human development affiliated with the Center for Multidisciplinary Research in Aging, Zusman Child Development Center, Division of Pediatrics and Community Health at the Ben Gurion University, Beer-Sheva, Israel, the medical director of the Division for Mental Retardation, Ministry of Social Affairs, Jerusalem, the founder and director of the National Institute of Child Health and Human Development. Has written numerous publications in the field of child health and human development, rehabilitation, intellectual disability, disability, health, welfare, abuse, advocacy, quality of life and prevention. Received the Peter Sabroe Child Award for outstanding work on behalf of Danish Children in 1985 and the International LEGO-Prize ("The Children's Nobel Prize") for an extraordinary contribution towards improvement in child welfare and well-being in 1987. E-Mail: jmerrick@internet-zahav.net. Website: www.nichd-israel.com
